# Diffusion deep learning for brain age prediction and longitudinal tracking in children through adulthood

**DOI:** 10.1162/imag_a_00114

**Published:** 2024-03-25

**Authors:** Anna Zapaishchykova, Divyanshu Tak, Zezhong Ye, Kevin X. Liu, Jirapat Likitlersuang, Sridhar Vajapeyam, Rishi B. Chopra, Jakob Seidlitz, Richard A.I. Bethlehem, Raymond H. Mak, Sabine Mueller, Daphne A. Haas-Kogan, Tina Y. Poussaint, Hugo J.W.L. Aerts, Benjamin H. Kann

**Affiliations:** Artificial Intelligence in Medicine (AIM) Program, Mass General Brigham, Harvard Medical School, Boston, MA, United States; Department of Radiation Oncology, Dana-Farber Cancer Institute and Brigham and Women’s Hospital, Harvard Medical School, Boston, MA, United States; Department of Radiology, Boston Children’s Hospital, Harvard Medical School Boston, MA, United States; Department of Psychiatry, University of Pennsylvania, Philadelphia, PA, United States; Department of Child and Adolescent Psychiatry and Behavioral Science, The Children’s Hospital of Philadelphia, Philadelphia, PA, United States; Lifespan Brain Institute, The Children’s Hospital of Philadelphia and Penn Medicine, Philadelphia, PA, United States; Department of Psychology, University of Cambridge, Cambridge, United Kingdom; Department of Neurology, Neurosurgery and Pediatric, University of California, San Francisco, San Francisco, CA, United States; Radiology and Nuclear Medicine, CARIM & GROW, Maastricht University, Maastricht, the Netherlands

**Keywords:** brain age, magnetic resonance imaging, regression diffusion models, deep learning, neuroimaging, brain volumetrics

## Abstract

Deep learning (DL)-based prediction of biological age in the developing human from a brain magnetic resonance imaging (MRI) (“*brain age*”) may have important diagnostic and therapeutic applications as a non-invasive biomarker of brain health, aging, and neurocognition. While previous deep learning tools for predicting brain age have shown promising capabilities using single-institution, cross-sectional datasets, our work aims to advance the field by leveraging multi-site, longitudinal data with externally validated and independently implementable code to facilitate clinical translation and utility. This builds on prior foundational efforts in brain age modeling to enable broader generalization and individual’s longitudinal brain development. Here, we leveraged 32,851 T1-weighted MRI scans from healthy children and adolescents aged 3 to 30 from 16 multisite datasets to develop and evaluate several DL brain age frameworks, including a novel regression diffusion DL network (AgeDiffuse). In a multisite external validation (5 datasets), we found that AgeDiffuse outperformed conventional DL frameworks, with a mean absolute error (MAE) of 2.78 years (interquartile range [IQR]: [1.2-3.9]). In a second, separate external validation (3 datasets), AgeDiffuse yielded an MAE of 1.97 years (IQR: [0.8-2.8]). We found that AgeDiffuse brain age predictions reflected age-related brain structure volume changes better than biological age (R^2^= 0.48 vs. R^2^= 0.37). Finally, we found that longitudinal predicted brain age tracked closely with chronological age at the individual level. To enable independent validation and application, we made AgeDiffuse publicly available and usable for the research community.

## 1 Introduction

The prediction of biological age from healthy brain magnetic resonance imaging (MRI) scans (i.e., “*brain age*”) has the potential for wide-ranging medical and scientific applications (Genon et al., 2022;Holm et al., 2022). Establishing reliable brain age prediction in large healthy-control populations would enable studying how various diseases, interventions, and socioeconomic factors influence brain development. When examined within cohorts affected by particular risk factors, the difference between predicted brain age and actual chronological age (i.e., “*brain age gap*”) may yield insights into how various external and internal factors affect brain development (Chen et al., 2022;Jawinski et al., 2022). Increased brain age gap has been associated with several brain disorders, such as schizophrenia, multiple sclerosis, mild cognitive impairment, and dementia (Kaufmann et al., 2019). Furthermore, accurately tracking the brain age gap may be useful in evaluating therapies designed to prevent neurocognitive disorder. Most research to this point has centered on adult and elderly conditions, where accelerated brain aging is inherently seen as a negative factor (Gaser et al., 2013). The implications of the brain age gap in developing children and young adults remain unclear, mainly owing to a lack of robust models that can accurately predict brain age out-of-sample (Erus et al., 2015). The existing brain age prediction models have limited generalizability because they fail to make accurate predictions on new datasets that differ from the data used for model training (Bzdok & Ioannidis, 2019).

Researchers have explored multiple approaches to brain age prediction, leading to a diverse set of methods with varying results (Tanveer et al., 2023). Direct comparison of these methods is challenging due to cross-study population differences, various imaging preprocessing techniques, and different evaluation strategies. Deep learning (DL) has emerged as a popular strategy for brain age prediction, given its remarkable success in trans-domain image analysis problems and its avoidance of time-consuming traditional feature extraction and preprocessing steps (Tanveer et al., 2023). Within pediatric or developing brain age prediction, there have been relatively few investigations (He et al., 2020;Hong et al., 2020;Hu et al., 2021;Mendes et al., 2021), likely due to limited data availability in this age range. Most existing studies demonstrate their models on single-institution datasets and have lacked multi-institutional external validations (He et al., 2020;Hong et al., 2020;Hu et al., 2021;Mendes et al., 2021), which is crucial for assessing true model generalization across diverse real-world settings and clinical utility. Factors including differences in scanners and protocols across sites, patient demographics, and other manifestations of dataset shift and drift are known to impact performance significantly (Bento et al., 2022;Ghafoorian et al., 2017). Furthermore, reviewing the pre-existing literature, we found no pediatric brain age models with implementable codes (He et al., 2020;Hong et al., 2020;Hu et al., 2021;Mendes et al., 2021), which is critical to moving the field forward and investigating these models’ clinical utility (Norgeot et al., 2020). Finally, brain age models developed from cross-sectional data may not be suitable for individual brain age tracking, and further study is needed to determine how brain age models perform across longitudinal time points, and their relationship to structural brain changes (Di Biase et al., 2023;Vidal-Pineiro et al., 2021).

In this study, we aim to address these gaps and develop a usable open-source model for reliable brain age prediction for childhood through young adulthood. Given the recent rise of generative DL methods (Han et al., 2022) and their promising results within the medical imaging domain (Yang et al., 2023), we developed a diffusion dual-guidance probabilistic regression model for pediatric brain age prediction (AgeDiffuse). We compared it to the state-of-the-art convolutional neural network (CNN) approaches, making this the first work, to our knowledge, to adapt diffusion models for image-based regression tasks. We demonstrate that diffusion-based models generalize well across two tiers of external validation, encompassing multi-institutional datasets from diverse geographic regions. We also investigate structural brain changes and their correlations with longitudinal brain age changes to yield interpretable insights into the model’s inner workings. Altogether, we present a robust model rigorously validated and made publicly available to the community, enabling the investigation of pediatric brain age in various clinical scenarios.

## 2 Materials and Methods

### 2.1 Dataset

**Fig. 1.**
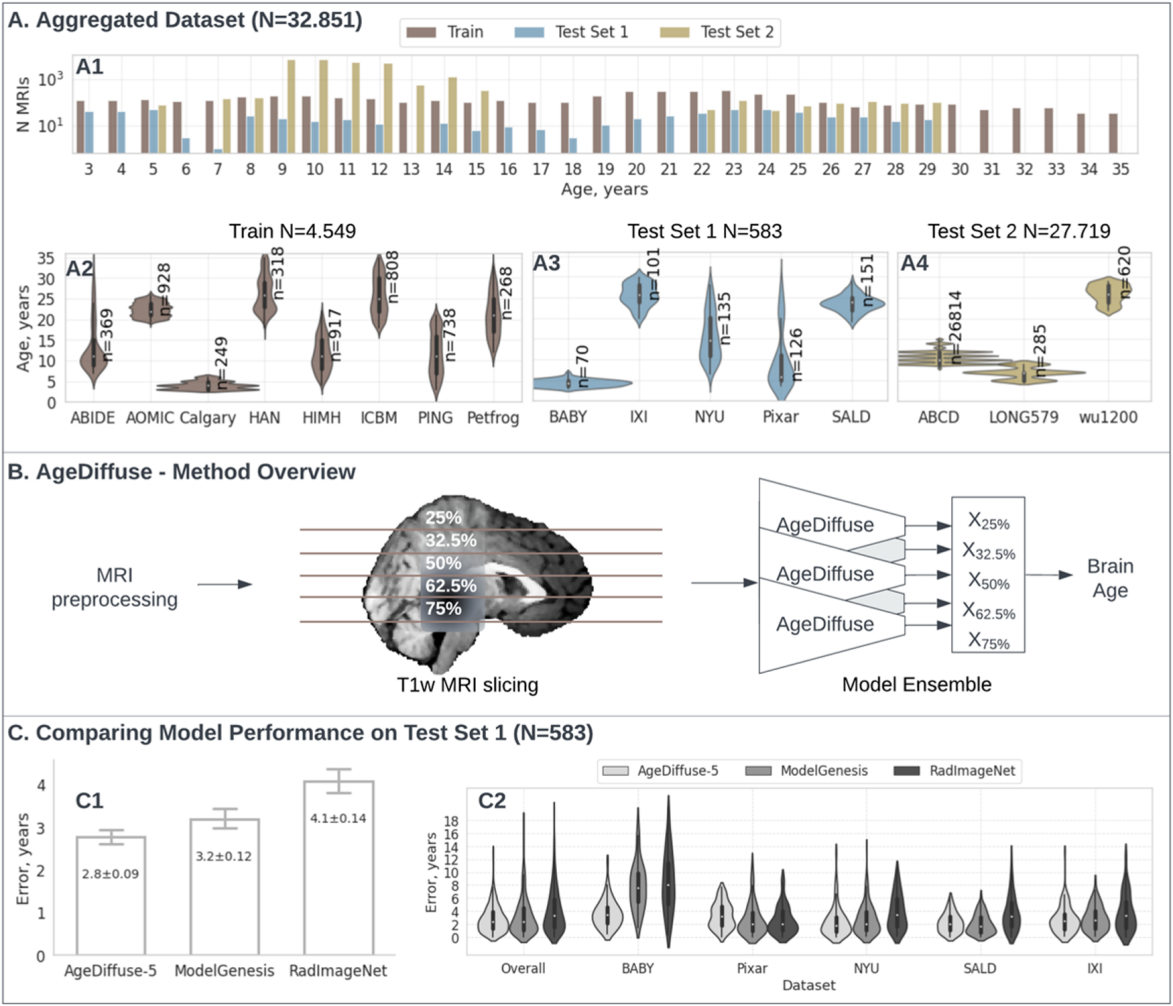
(A) Aggregated dataset overview (total N = 32,851). (A1) Bar plot with number of MRI T1w per age group in Train (N = 4,549)/Test Set 1(N = 583)/Test Set 2(N = 27,719); the y-axis is log scaled. (A2-A4) Violin plots for dataset age distributions in Train (A2)/Test Set 1 (A3)/Test Set 2 (A4). The violins represent kernel density estimates of the age distribution in each dataset. Wider sections of the violins indicate a higher probability density at that error level. (B) AgeDiffuse method overview: MRI preprocessing, 2D slice selection, AgeDiffuse model prediction, and model ensembling. (C) Model performance comparisons on Test Set 1 (N = 583; 5 datasets). (C1) Bar plot for model-wise mean comparison in Test Set 1, with 95% confidence intervals overlay. The diffusion 5-slice ensemble (AgeDiffuse-5) performed with the highest accuracy among all models with mean error 2.8 years[IQR = 1.3-3.9] compared to ModelGenesis mean error 3.2 years [IQR = 1.0-4.5] and RadImageNet mean error 4.1 years [IQR = 1.5-5.8]. (C2) Violin plots for model-wise error distribution comparison in Test Set 1. MR = Magnetic resonance imaging, AgeDiffuse = Novel regression dual-guidance diffusion model for brain age prediction.

We curated T1w MRIs without contrast enhancement from 16 datasets and stratified them by age so that each age had 100 scans per year maximum in the training set, to avoid data imbalance during the training (ABCD (Casey et al., 2018), ABIDE (Di Martino et al., 2017), AOMIC (Snoek et al., 2021), Baby Connectome (Howell et al., 2019), Calgary (Reynolds et al., 2020), ICBM (Kötter et al., 2001), IXI (*IXI Dataset – Brain Development*, n.d.), NIMH (Evans, 2006), PING (Jernigan et al., 2016), Pixar (*MRI Data of 3-12 Year Old Children and Adults during Viewing of a Short Animated Film*, n.d.), SALD (Wei et al., 2018), NYU2(CoRR) (Zuo et al., 2014), Healthy Adults (Nugent et al., 2022); Long579(*OpenNeuro*, n.d.), WU1200(Van Essen et al., 2013); seeSupplementary Material A5. To create robust train and test sets, we divided the data into training, validation, and test sets using a rough 70/15/15 split. When splitting the data, we matched the age distribution coverage between the training and test sets as closely as possible. This ensured that both sets had similar representation across the full range of ages. At the same time, we preserved the integrity of each original dataset by keeping all subjects from a given source together in either the training or test set. This avoided contaminating the test data with subjects from datasets used in training. The training data consisted of 8 datasets totaling 4,549 subjects (Fig. 1, Panel A2). We held out 5 separate datasets with 583 total subjects as our first test set (Fig. 1, Panel A3). We also created a larger second test set using 3 primary datasets with 27,719 subjects (Fig. 1, Panel A4).

### 2.2 Image preprocessing and registration

Scans were co-registered to MRI age-dependent T1-weighted asymmetric brain atlases, generated from the NIH-funded MRI Study of Normal Brain Development (hereafter, NIHPD, for NIH pediatric database (Fonov et al., 2011)) with rigid registration using SlicerElastix (Lasso, 2023) (Elastix generic rigid preset). All MRIs were skull-stripped using HD-BET (Isensee et al., 2019). MRI images were rescaled to 1-mm isotropic voxel size to preserve anatomical size differences using the itk-elastix Python package (*ITKElastix*, 2023). N4 bias field correction was performed using the simple-itk Python library. We then normalized MRI images, performed median filtering, removed background pixels using Otsu filtering, and standardized the intensity scale. After preprocessing, we identified axial slices with at least 1% non-zero voxels to ensure consistent anatomical coverage across subjects. We extracted five equidistant percentile slices from these valid slices along the inferior-superior axis—the 25th, 37.5th, 50th, 62.5th, and 75th percentiles. The 50th percentile median slice focused on central structures, while lower and higher percentile slices sampled inferior and superior regions. This multi-slice approach provided an anatomically distributed sampling of the pediatric brain for 2D deep learning analyses.

### 2.3 Regression dual-guidance diffusion model

The overall pipeline is shown inFigure 1B. We adapt the dual-guidance diffusion model architecture for medical image classification (DiffMIC) proposed byYang et al. (2023). DiffMIC is a novel diffusion probabilistic model for robust medical image classification. It introduces a Dual-Granularity Conditional Guidance (DCG) strategy that provides global and local priors to guide the diffusion process. DCG helps distinguish critical tissues and lesions at both whole image and regional levels. DiffMIC also enforces Condition-specific Maximum Mean Discrepancy (MMD) regularization to ensure consistency between the predicted noise distributions and targets for each condition to capture mutually relevant information. At its core is a UNet denoising model that leverages noisy image embeddings and dual priors to predict the noise distribution in each diffusion step. It is trained end-to-end with noise estimation and MMD regularization losses. Specifically, we altered the dual-granularity conditional guidance (DCG) model to be optimized with a mean-squared error (MSE) loss between the predicted and ground truth age labels. This tailors the guidance model to provide informative global and local priors for denoising in the diffusion process.

During training, the diffusion model utilizes a conventional DDPM training approach. The diffusion time step “t” is chosen from a uniform distribution ranging from 1 to T, and the noise is scheduled linearly with β_1_set to 1 × 10^-4^and β_T_set to 0.02. The image encoder is implemented as ResNet18.

The condition-specific Maximum-Mean Discrepancy (MMD) regularization losses are used to learn mutual information between sampled noise and the Gaussian distribution. The regularization is applied to global and local priors, ensuring faster, stable convergence by preserving mutual information and enhancing dual-prior feature representations. The total loss${ℒ}_{diff}$of the AgeDiffuse network is defined as follows:

$$\begin{array}{c} {ℒ}_{diff}={ℒ}_{e}+\frac{1}{2}\left({ℒ}_{MMD}^{g}+{ℒ}_{MMD}^{l}\right) \end{array}$$ (1)

The noisy variable$y_{t}$is sampled in the diffusion process based on global and local priors. The raw image data for the global stream are input into the global encoder*τ_g_*, followed by a 1 × 1 convolutional layer to generate a saliency map for the entire image. The global prior${\hat{y}}_{g}$is then predicted by averaging the responses obtained from the entire saliency map. In the case of the local stream, the Regions of Interest (ROIs) are isolated based on their significant responses in the saliency map of the entire image. Each ROI is then processed through the local encoder*τ_l_*to acquire a feature vector. Subsequently, a gated attention mechanism (Ilse et al., 2018) is employed to combine all feature vectors from the ROIs, producing a weighted vector. This weighted vector is then used to compute the local prior${\hat{y}}_{l}$through a linear layer. The noise estimation loss${ℒ}_{e}$is defined as follows:

$$\begin{array}{c} {ℒ}_{e}={\|\in -\in_{\theta }\;\left(\rho (x),\;y_{t},\;{\hat{y}}_{g},\;{\hat{y}}_{l},\;t\right)\|}^{2} \end{array},$$ (2)

where#~N(0,I),ρ(x)is an image feature embedding,${\hat{y}}_{g}$is a global prior,${\hat{y}}_{\text{l}}$is a local prior, and${\text{y}}_{\text{t}}$is a predicted noise at timestamp t.

Finally, a fully connected layer predicts the noise with an output dimension of one for the regression task.

Additionally, we added an early stopping rule with patience = 50. We trained all models separately on an A6000 Nvidia GPU; details on DiffMiC implementation can be found in the paper (Yang et al., 2023); technical details and code can be found on the GitHub repository (https://github.com/AIM-KannLab/pediatric-brain-age).

### 2.4 Model ensembling

**Fig. 2.**
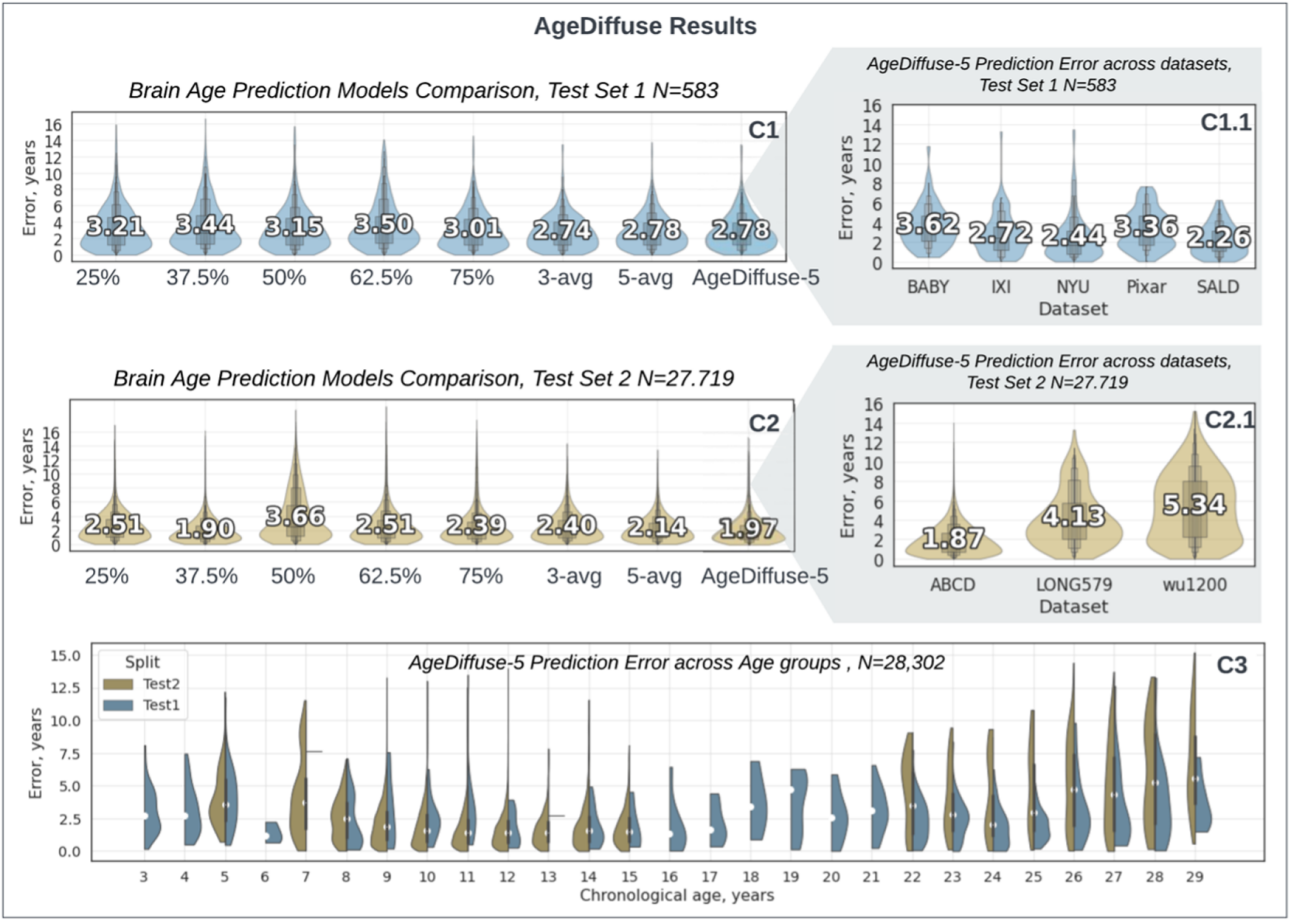
Violin plots for AgeDiffuse brain age prediction in developing children: dual-tiered external validation with text median overlays. Violin plots for slice-wise diffusion-based model comparison on (C1) Test Set 1(N = 583, 5 datasets) and (C2) Test Set 2 (N = 27,719, 3 datasets). The violins represent kernel density estimates of the error distribution with a text overlay of mean values. Wider sections of the violins indicate a higher probability density at that error level. The diffusion 5-slice ensemble (AgeDiffuse-5) consistently performed with the highest accuracy among all models on both test sets (C1.1-C2.1). (C3) Violin plots for prediction error distribution for each chronological age, divided by Test Set 1/Test Set 2. AgeDiffuse-5 demonstrated strong performance across the age range, with mild performance degradation for subjects older than 25 years (SeeSupplementary Fig. S1 and Supplementary Material A4. Outlier Analysis).

We conducted experiments comparing simple model averaging with varying ensemble sizes and outlier exclusion to evaluate different ensembling techniques for improving predictive uncertainty. Ensembles of sizes 3 and 5 were constructed by training identical model architectures for different slice quantiles. We investigated an “outlier exclusion” ensembling technique to mitigate the effect of outlier scans on age prediction. We hypothesized that these outliers were likely due to image artifacts, poor quality scans, MRI registration, or other out-of-distribution characteristics. For the outlier exclusion ensemble, five models were trained, and each model produced a brain age prediction for a given input. The standard deviation of the predictions from the five models was calculated. Any individual model prediction that was an outlier, meaning it deviated more or less than one standard deviation from the ensemble average, was excluded. The remaining model predictions were averaged to produce the final consensus prediction. All ensembles were evaluated by two-tiered external validation (Fig. 2).

### 2.5 Brain substructure volumetrics

We used the centile definition described inBethlehem et al. (2022); for details on the normative growth charts, please refer to the original publication. We obtained a total of 25.097 overlapping scans from datasets ABCD (Casey et al., 2018), IXI (*IXI Dataset – Brain Development*, n.d.), Pixar (*MRI Data of 3-12 Year Old Children and Adults during Viewing of a Short Animated Film*, n.d.), SALD (Wei et al., 2018), and WU1200 (Van Essen et al., 2013); seeSupplementary Material A5. Four key volumetric centile measurements (WMV, GMV, sGMV, VV) were compared pairwise between “older”/”younger” and “average” age groups for each gender. Brain age gap was defined as predicted brain age minus chronological age. “Younger” brain age gap was defined as predicted brain age >1 standard deviation below the mean; “Older” brain age gap was defined as predicted brain age >1 standard deviation above the mean. The “average” group was defined as those subjects whose brain age gap lies within one standard deviation. Pairwise Mann-Whitney U tests were used to compare the older group to the average age group for each volumetric and gender. Bonferroni correction was applied to adjust for multiple comparisons (adjusted alpha = 0.05/16 = 0.003125). Cohen’s d effect sizes were calculated to quantify the standardized mean difference between groups for each volumetric and gender.

### 2.6 Longitudinal brain age analysis

To calculate the association between longitudinal changes in brain age and brain volume over time in 1,392 participants, we calculated the rate of volumetric measures change (WMV, GMV, sGMV, VV) for each time point and each subject and calculated their brain age using AgeDiffuse-5. The acceleration values were then categorized as “Accelerated,” “Decelerated,” or “Stable” based on standard deviation thresholds. For each volumetric, pairwise two-sided Mann-Whitney U tests compared the “Stable” group to “Accelerated”/“Decelerated.” Bonferroni correction was applied to adjust for multiple comparisons across the four volumetrics (P < 0.006).

### 2.7 Performance evaluation and statistical analysis

The primary endpoint was the mean average absolute error of predicted age compared to chronological age (ground truth). Violin and box plots with median errors were used for visual comparison. Associations between substructures and brain age or chronological age were evaluated with multivariable logistic regression. Model goodness of fit was evaluated by comparing R^2^values (SeeSupplementary Material A2). Pairwise tests for significance were based on the two-sided Mann-Whitney U-test, and P values were adjusted for multiple comparisons using the Bonferroni correction.

## 3 Results

### 3.1 Diffusion regression for brain age

We aggregated a dataset with 32,851 MRI T1-weighted (T1w) scans (Train Set N = 4,549, Test Set 1 N = 583, Test Set 2 N = 27,719) from subjects aged 3-30 years from 16 publicly available, multisite datasets of healthy, developing children through adulthood (Fig.1A; Methods “Dataset” section). We then developed an MRI preprocessing and registration pipeline (Fig. 1B, see Methods “Image Preprocessing and Registration” section). We evaluated the performance of two state-of-the-art DL approaches for medical imaging: 1) a medical-domain, pretrained 2D convolutional neural network (RagImageNet (*RadImageNet: An Open Radiologic Deep Learn-ing Research Dataset for Effective Transfer Learning | Radiology: Artificial Intelligence*, n.d.)) and 2) a self-supervised, pretrained 3D UNet (ModelGenesis (Zhou et al., 2019)) (seeSupplementary Material A1. Model hyperparameter tuning). We then developed a 2D diffusion-based regression model, called AgeDiffuse model, that uses dual-granularity guidance and condition-specific maximum mean discrepancy (MMD) regularization. AgeDiffuse was adapted from a dual-guidance diffusion model for medical image classification (Yang et al., 2023) (see Methods “Regression Dual-Guidance Diffusion Model”). Dual-guidance models use both global and local priors for conditional guidance at each step, and have the advantage of modeling representations with both holistic and fine-grained understanding of medical images.

On initial multi-institutional external validation (Test Set 1, N = 583, 5 datasets), the diffusion network using the median axial slice as input (AgeDiffuse-1) achieved the highest accuracy compared to other methods for predicting chronological age (Table 1., MAE = 3.15 years, IQR = [1.27-4.41]). To investigate if sampling from multiple axial slices would improve model performance, we trained 2D diffusion models on axial MRIs sampled from the 25, 27.5, 50 (median), 62.5, and 75 percentile slices in the craniocaudal distribution and then tested model ensembling across slices (see Methods “Model Ensembling”). The 5-slice diffusion network ensemble (AgeDiffuse-5) achieved the highest accuracy with MAE = 2.78 years (IQR = [1.24-3.92]) outperforming 3D approach ModelGenesis MAE = 3.19 years (IQR = [1.0-4.5]) and 2D RadImageNet MAE = 4.07 years ([IQR = 1.5-5.8]). To further test the model generalizability, we conducted a blinded secondary validation on three external datasets (Test Set 2; N = 27,719). We compared simple model averaging with varying sizes and outlier exclusion to evaluate different ensembling techniques and found that the five-slice AgeDiffuse-5 model yielded the best brain age prediction with MAE = 1.97 years (IQR = [0.76-2.75]) (Fig. 2). For all models, accuracy decreased for later ages, particularly over 25 years old, though AgeDiffuse had less performance degradation than other models (SeeSupplementary Fig. S1andSupplementary Material A4. Outlier Analysis).

**Table 1.** Comparison of mean absolute error (MAE) between different models on Test Set 1.

Method	2d/3d	MAE, years [IQR]
RadImageNet	2D – median slice	4.07 [1.5-5.8]
ModelGenesis	3D	3.19 [1.0-4.5]
AgeDiffuse-1	2D – median slice	3.15 [1.27-4.41]
AgeDiffuse-5	2D Model ensemble: 25th, 37.5th, median, 62.5th, 75th slices	2.78 [1.24-3.92]

2D equidistant quantile slices ensembling (AgeDiffuse-5) provides a robust prediction while being less susceptible to noise and outperforms other methods.

IQR = interquartile range.

Recent studies have proposed bias correction for deep learning regression models given the tendency for models to underestimate older age and overestimate younger age (de Lange & Cole, 2020), albeit this correction strategy is controversial (Butler et al., 2021). We investigated brain age bias correction and found that it did not improve prediction accuracy (SeeSupplementary Material A3. Age-Bias Correction).

### 3.2 Brain age and brain structure volumes

**Fig. 3.**
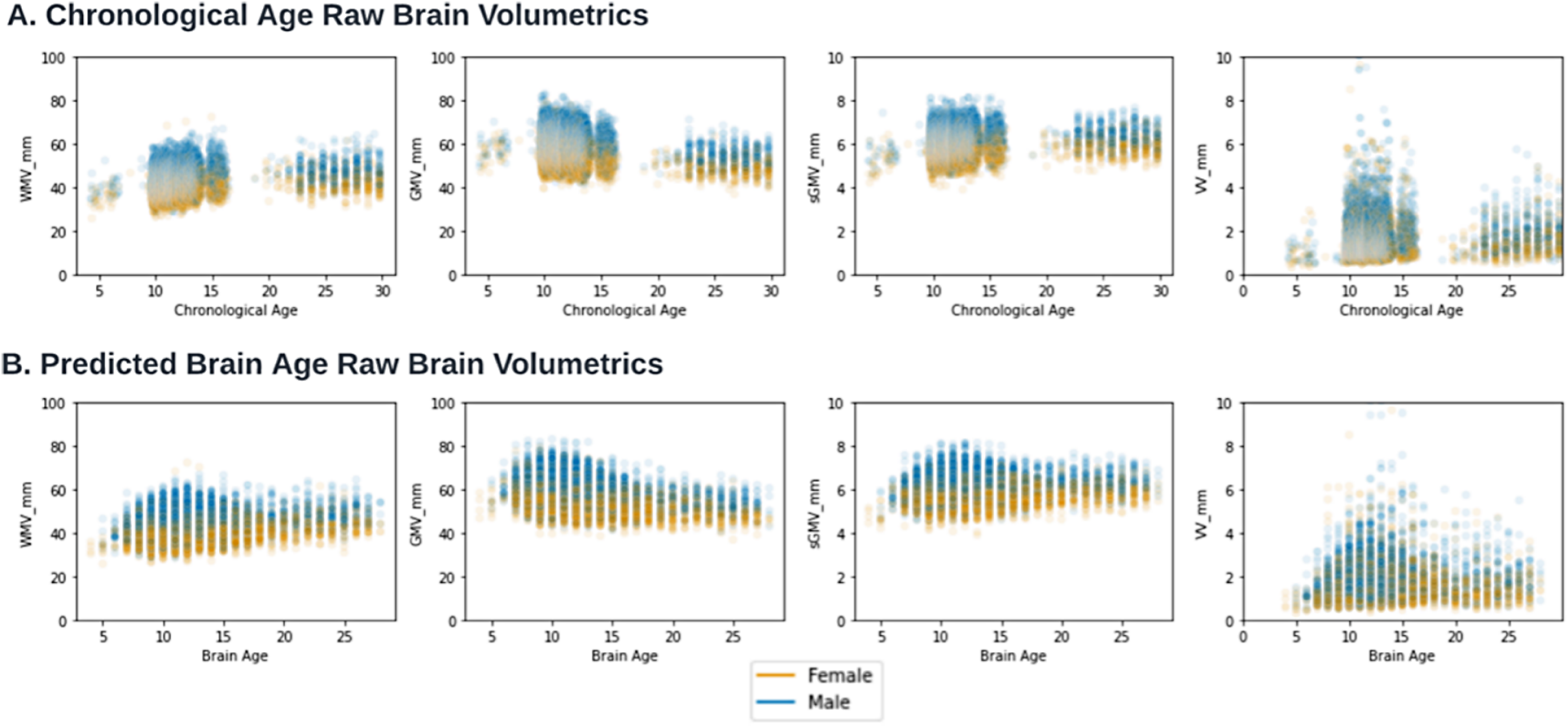
(A). Deep Learning Brain Age and Structural Tissue Volumes. Brain structural tissue volumes for white matter (WMV), grey matter (GMV), total subcortical grey matter volume (sGMV), and ventricles (VV) are plotted for each cross-sectional control scan as a function of (A) chronological age and (B) predicted brain age using AgeDiffuse-5.

Interpretability of deep learning algorithms is clouded by the black-box nature of hidden layers (Castelvecchi, 2016), and brain age models to date have not investigated the underlying biological and anatomical bases of predictions. To improve the understanding of underlying factors contributing to brain age prediction, we analyzed associations with brain substructure volumes derived fromBethlehem et al. (2022)within overlapping patients from both studies for (N = 25,096, age mean 12.2,Fig. 3). We found that, graphically, chronologic age and predicted brain age had similar associations with brain substructure changes over development. We then examined how brain age gap, defined as per Eq (3), is associated with brain substructure volumes.

$$\begin{array}{l} Brain\;age\;gap=Brain\;age\;predicted\\ \;\;\;\;\;\;\;\;\;\;\;\;\;\;\;\;\;\;\;\;\;\;\;\;\;\;\;\;\;\;\;\;\;\;\;\;\;\;\;\;\;–chronological\;age \end{array}$$ (3)

**Fig. 4.**
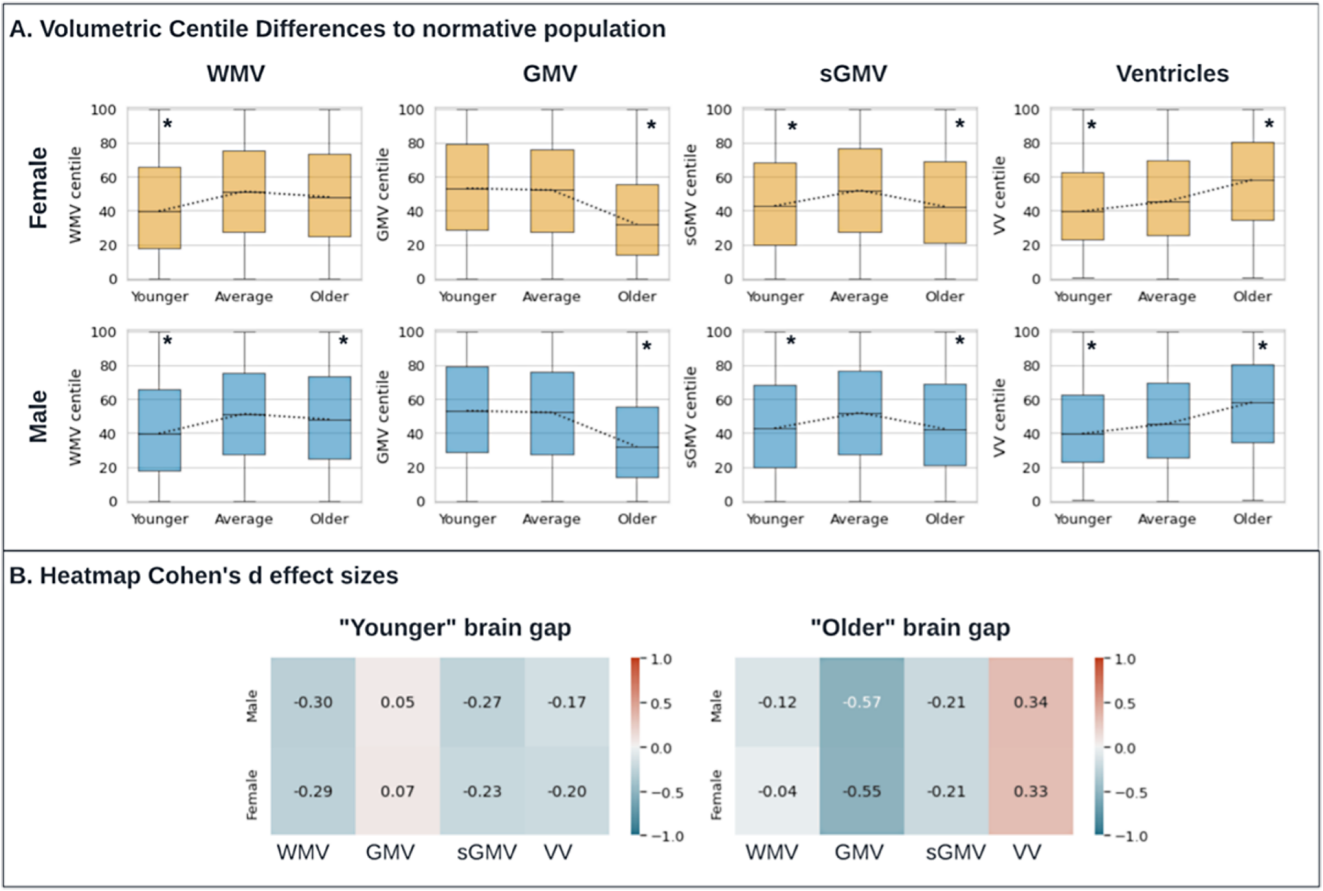
(A) Box plots for brain age gap and association with brain substructure volumes. Brain age gap was defined as predicted brain age minus chronological age. “Younger” brain age gap was defined as predicted brain age >1 standard deviation below the mean; “Older” brain age gap was defined as predicted brain age >1 standard deviation above the mean. The “average” group was defined as those subjects whose brain age gap lies within one standard deviation. Pairwise tests for significance were based on the Mann-Whitney U-test, and P values were adjusted for multiple comparisons using the Bonferroni correction. Significant differences (with corrected P < 0.003) are highlighted with an asterisk. (B) Heatmap of Cohen’s d effect sizes comparing brain age outliers versus within normal range, stratified by gender and key volumetric measures from MRI. VV = cerebrospinal fluid, WMV = white matter volume, GMV = gray matter volume, sGMV = total subcortical grey matter volume.

Specifically, we investigated whether “younger brain” and “older brain” outliers, defined as predicted brain age >1 standard deviation above or below the mean prediction for a given chronological age and sex, were associated with brain substructure volumes. We found that younger brain outliers had increased gray matter volume (GMV) and decreased white matter volume (WMV) and ventricle volume (VV), and older brain outliers had decreased subcortical gray matter volume (sGMV) and GMV, and increased VV (Mann-Whitney U test < 0.003 for each,Fig. 4A). Effect sizes were largest for VV and GMV for “older brain” (Cohen’s*d* > 0.2,Fig. 4B).

To determine how brain substructure volume was comparatively associated with chronological versus brain age, we compared two multivariable linear regression models with brain substructure volumes and sex as independent variables and chronological age or predicted brain age as dependent variables. We found that brain substructure volume was more associated with brain age than chronological age (R^2^0.37 vs. R^2^0.47; SeeSupplementary Material A2. Linear model diagnostics).

### 3.3 Longitudinal brain age evaluation

**Fig. 5.**
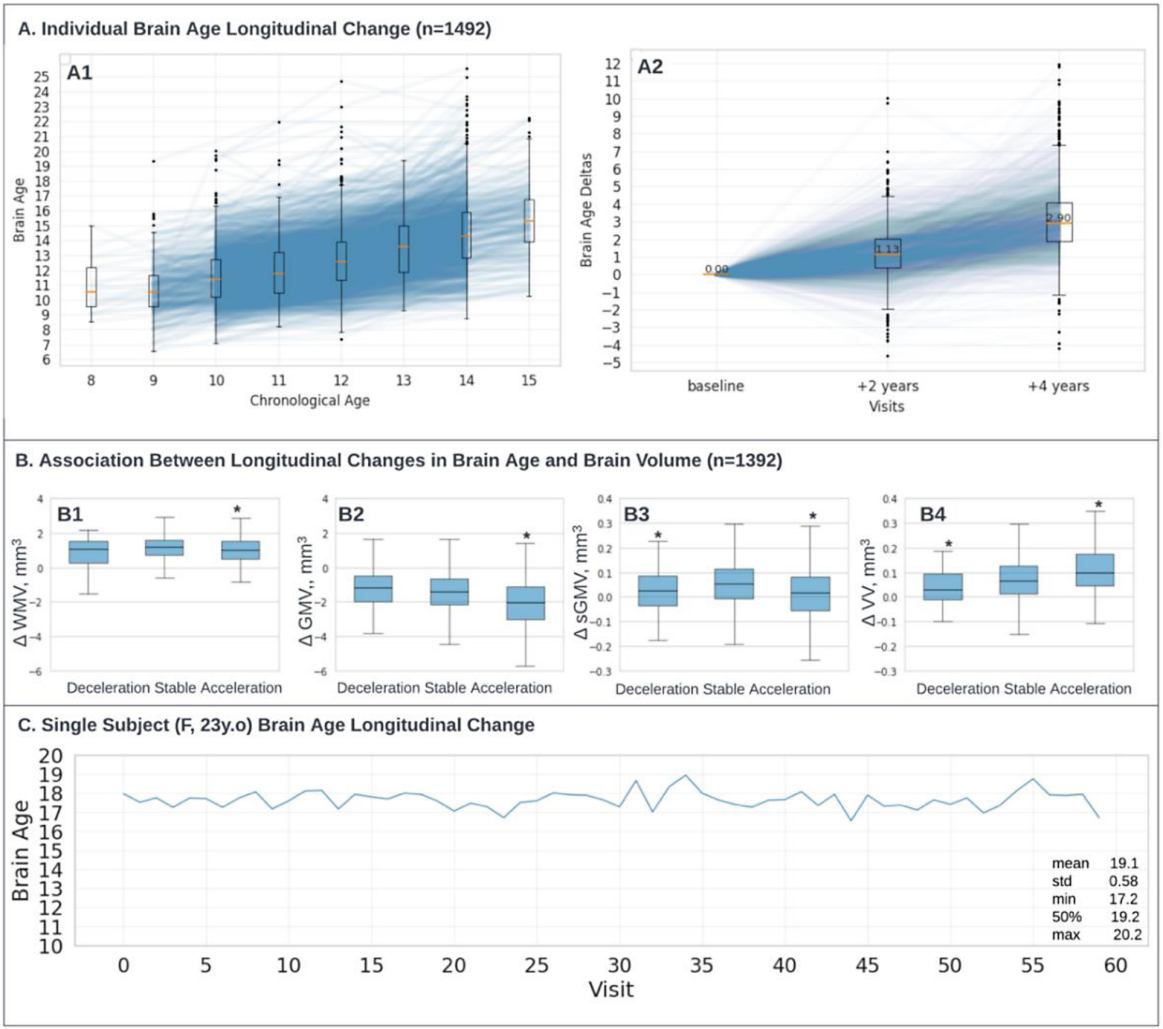
(A) Individual brain age longitudinal change (n = 1,492). (A1) Individual lines show brain age longitudinal change for 1,492 subjects (ABCD dataset (Casey et al., 2018)) who had three visits within 2 years in between with boxplot overlay. (A2) Individual brain age changes in-between visits: baseline, +2 years timestamp was computed as the brain age difference for each subject between the second and baseline visit; +4 years timestamp was computed as the brain age difference for each subject between the third and baseline visit. (B) Association Between Longitudinal Changes in Brain Age and Brain Volume (n = 1,392). The study examined the relationship between changes in brain age and changes in brain volume over time in 1,392 participants. Significant volumetric variables are marked with an asterisk (P values were adjusted for multiple comparisons, P < 0.006, See Methodology “Longitudinal Brain Age Analysis”) (C). The participant (Female, 23 years: 28andme dataset*(*Pritschet et al., 2020)) underwent daily testing for two studies of 30 consecutive days with one year in between (60 scans in total). The mean predicted brain age is 19.1 years, with a standard deviation of 0.58. VV = cerebrospinal fluid, WMV = white matter volume, GMV = gray matter volume, sGMV = total subcortical grey matter volume.

A barrier to the clinical utility of brain age models is that, due to data availability, models are developed on cross-sectional data, yet the clinical impact would be strengthened by the ability to track individual brain age over time (and how exposures modify individual-level brain age). There is concern that brain age prediction derived from cross-sectional data does not generalize to individual-level brain age change (Vidal-Pineiro et al., 2021). To investigate this, we applied AgeDiffuse-5 to longitudinal data available within the ABCD dataset, where each subject contains 3 MRI time points at roughly 2-year intervals. On longitudinal analysis, we found that predicted brain age tracked directionally with chronologic age, with a slight underestimation of chronological age that was within the margin of algorithm expected prediction error (Fig. 5A).

We further examined the relationship between changes in brain age and brain substructure volumes over time in 1,392 subjects with available data. We found that the rate of change in brain age between subsequent MRI timepoints was associated with the rate of changes in brain substructure volumes over the same time interval (Fig. 5B). Accelerated brain aging (i.e., change >1 standard deviation above the mean) was associated with an increased rate of growth in VV and a decreased rate of growth in sGMV, GMV, and WMV. Decelerated brain aging (i.e., change <1 standard deviation above the mean) was associated with a decreased rate of growth in sGMV and VV (adjusted P < 0.006,Fig. 5B, see Methods “Longitudinal Brain Age” section).

Finally, to demonstrate the stability of brain age predictions at the intra-patient level, we applied AgeDiffuse-5 for a single female participant tested over 60 days with daily MRI across two studies one year apart (28andme dataset (Pritschet et al., 2020)). The mean predicted brain age was 19.1 years with a standard deviation of 0.58 across (Fig. 5C). The low standard deviation indicates consistent predictions across the 60 test days, with no observable trends in predicted age or error over time.

## 4 Discussion

Imaging-based brain age prediction in developing humans may have far-reaching clinical applications, though clinical translation has been limited by small datasets, unclear generalizability, and lack of reproducible models. In this study, we aggregated the largest to-date dataset of MRI scans for children through adulthood to develop and rigorously validate a diffusion-based regression neural network (AgeDiffuse) for brain age prediction. We found that AgeDiffuse, ensembled over multiple MRI slices among scans from a multi-institutional repository, demonstrated highly accurate and generalizable brain age prediction, outperforming current state-of-the-art models. AgeDiffuse was subject to two-tier validation across multiple datasets, and implementable code has been released open source as a resource for the scientific and clinical communities. Our results show that ensembling across axially sampled MRI slices can improve performance and that a technique where slice-based outlier predictions are excluded before averaging improves generalizability. Such a technique could enable accurate brain age prediction in patients with focal brain pathologies (e.g., tumors, vascular malformations, stroke), as the model would exclude slices with aberrant prediction. Additionally, we found that application of AgeDiffuse to longitudinal data was reliable and that the brain age prediction was driven, in part, by interpretable brain substructure volume changes that are associated with development. We believe this model is positioned for investigation in various pediatric conditions to track and predict brain development and neurocognitive outcomes in various diseases (e.g., brain tumors, endocrine dysfunction) and/or interventions (e.g., radiation therapy, hormonal therapies) that may affect normal development and neurocognitive outcomes.

Brain age tracking may reveal clinically relevant states, such as changes in the neurocognition (Kaufmann et al., 2019), that could guide interventions and triage patients for escalated care. Previous studies have linked the brain age gap to various biomedical factors and lifestyle variables in healthy cohorts (Anatürk et al., 2021;Cole et al., 2019;*Multimodal Image Analysis of Apparent Brain Age Identifies Physical Fitness as Predictor of Brain Maintenance | Cerebral Cortex | Oxford Academic*, n.d.). Large-scale datasets have recently enabled the development of normative growth charts for key structural MRI metrics across ages, providing an essential reference for quantifying individual variation (Bethlehem et al., 2022). These brain charts identify neurodevelopmental milestones, show reliability across scans, and can benchmark deviations in disorders. In this study, for the first time, we demonstrate that DL brain age prediction is associated with substructure volume changes that signify age-related atrophy at the individual-level. Our findings suggest that DL brain age and substructure volumetrics are likely complementary measures, though additional research should examine how much incremental information is added by DL brain age compared to structural volumetrics when predicting neurocognitive endpoints.

In the context of children and developing humans in the early part of the lifespan, several DL methods have emerged for age inference directly from 3D images, eliminating the need for prior feature extraction (Tanveer et al., 2023). Mendes et al (Mendes et al., 2021) achieved an average 10-fold average Mean absolute error (MAE) of 1.57 years using 3D VGG16, utilizing data from two public datasets (ABIDE-II, N = 580, and ADHD-200, N = 922) covering an age range of 6 to 20 years. He et al (He et al., 2020) compared the performance of 2D-ResNet18+LSTM and 3D neural networks, reporting an MAE of 1.14 years versus 2.64 years on an external cohort with subjects aged 0 to 6 years (private dataset, N = 428). Hong et al. (Hong et al., 2020) MAE of 67.6 days on an internal held-out test set of 44 subjects aged 0 to 5 years, utilizing a 3D CNN approach. Additionally, Hu et al. (Hu et al., 2021) proposed a 3D CNN model, demonstrating an average MAE of 1.01 years in a 5-fold cross-validation on 880 subjects (ABIDE I and II, ADHD200), spanning ages 6 to 18 years. However, only one of these methods has a publicly available code with no model weights publicly available (Hong et al., 2020), and none have compared model generalization across multiple studies that were not included in the model training process. The focus on narrow age ranges and lack of rigorous evaluation on heterogeneous public datasets raises questions about model generalizability and reproducibility. While we were not able to directly benchmark AgeDiffuse to the models due to a lack of implementable code, we utilized three comparison approaches with similar, established 2D and 3D CNN architectures and optimized them with transfer and self-supervised learning. We found that diffusion-based model performance—even without ensembling—had improved performance. We hypothesize that the brain age correction procedure does not generalize well on unseen datasets and does not capture the non-linear, complex relationship between brain age and chronological age, unlike deep learning.

Our study highlights the challenges of brain age model generalization and has several important limitations. We noted that brain age prediction tends to become less precise in older age ranges, likely due to developmental and environmental heterogeneity (Bashyam et al., 2020;Cole & Franke, 2017;*Structural and Functional MRI Data Differentially Predict Chronological Age and Behavioral Memory Performance | eNeuro*, n.d.). Specifically, we observed a performance drop in one of the smaller external validation datasets (WU1200), with an age range of 22–29 years. Notably, this population also had differences in substructure volumetrics, indicating that the performance drop may be due more to true population differences than problems with the model (Supplementary Material A4). These findings have been noted previously (Bashyam et al., 2020;Cole & Franke, 2017;*Structural and Functional MRI Data Differentially Predict Chronological Age and Behavioral Memory Performance | eNeuro*, n.d.) and have implications for the utility of brain age in older populations. They also suggest that individual-level longitudinal trajectories of brain age may be more informative than snapshots compared to a general population. We were able to establish feasibility of longitudinal analysis within the ABCD cohort, although this was limited to age ranges 8–16, and further work is ongoing to evaluate longitudinal changes over longer intervals. Secondly, the aggregated MRI dataset might have a bias towards North American and European populations. This is a common pitfall of healthcare inequity that must be addressed by increasing the number of studies in other demographics. Moving forward, curating test sets that capture wide pediatric age ranges and those with real-world clinical data will better assess model performance for diverse real-world utilization, and we would recommend pilot testing in underrepresented patient groups prior to implementation (de Lange et al., 2022). Additionally, utilizing multiple imaging modalities (e.g., T1w and T2) could help to refine model prediction further.

Although accurately predicting age is important, recent research indicates a discrepancy between the usefulness of a model and its level of accuracy (Jirsaraie et al., 2023). Our future work will involve gathering additional data for researching cognitive outcomes and examining their correlation with AgeDiffuse predictions.

Lastly, as with many DL models, AgeDiffuse might suffer from the “black box” issue, making it challenging to interpret how specific features influence age predictions. We attempted to provide a measure of interpretability to our model predictions by evaluating volumetric structural correlations between predicted brain age and chronological age (seeSection 3.2. Brain age and brain structure volumes) and found that AgeDiffuse brain age predictions reflected age-related brain structure volume changes better than biological age.

## 5 Conclusions

In this work, we developed and rigorously validated an accurate brain age prediction model, AgeDiffuse, for children through adulthood using diffusion regression on multiple datasets. We demonstrated that this approach could be feasibly applied to longitudinal data to track individual brain age changes over time. Further analyses suggested that deep learning brain age and substructure volumetrics carry complementary information. With this study, we release, to our knowledge, the first fully implementable deep learning brain age algorithm to the scientific community. Independent validation of our model in the context of various conditions with longitudinal cohorts and clinical endpoints is needed to maximize the impact of deep learning-based brain age prediction for children through adulthood.

## Supplementary Material

Supplementary Material

## Data Availability

The complete dataset (Supplementary Material A5) aggregated for this study contains primary datasets that differ widely in terms of their “openness,” that is, their availability for secondary use without restrictions or special efforts by the primary study team. Preliminary studies ranged from fully open and downloadable datasets in the public domain to more restricted datasets that could only be used for specific purposes, under separate agreements, or after special efforts had been made to provide data in shareable form. The model training and testing code is available at Zenodo repository 10.5281/zenodo.10728314.
