# Supplementary material for: Diffusion deep learning for brain age prediction and longitudinal tracking in children through adulthood
Source: Imaging Neurosci (Camb). 2024 Mar 25;2:imag-2-00114. doi: 10.1162/imag_a_00114 (PMC12247581; doi:10.1162/imag_a_00114)
Supplement: Supplementary Material [file imag_a_00114-supp.pdf]

Supplementary Material for
“Diffusion Deep Learning for Brain Age Prediction and Longitudinal Tracking in Children Through Adulthood“

A1. Model hyperparameter tuning

RagImageNet Finetuning

The RadImageNet database is an open-access medical imaging database. It was designed to improve transfer learning performance on downstream medical imaging applications(*RadImageNet: An Open Radiologic Deep Learning Research Dataset for Effective Transfer Learning | Radiology: Artificial Intelligence*, n.d.). We used RadImageNet pretrained ResNet50 backbone and added 3 fully connected layers (sizes: 1024, 128, 1) in combination with dropout layers (0.5) and fine-tuned unfreezing all layers using Adam optimizer learning rate 1e-3 that reduces on plateau and MAE loss, with an early stopping rule (patience=10) and batch size 32.

ModelGenesis Finetuning

We pre-trained ModelGenesis 3D U-net backbone in a self-supervised way on the brain MRI scans as described in (Zhou et al., 2019). We further used the encoder with one fully connected layer (size:512) for finetuning using SGD optimizer with learning rate 1e-5 that reduces on plateau for 20 epochs and MSE loss. We used batch size 1 and downscaled MRI T1w to [64,64,64] patch size with resolution [2,2,2].

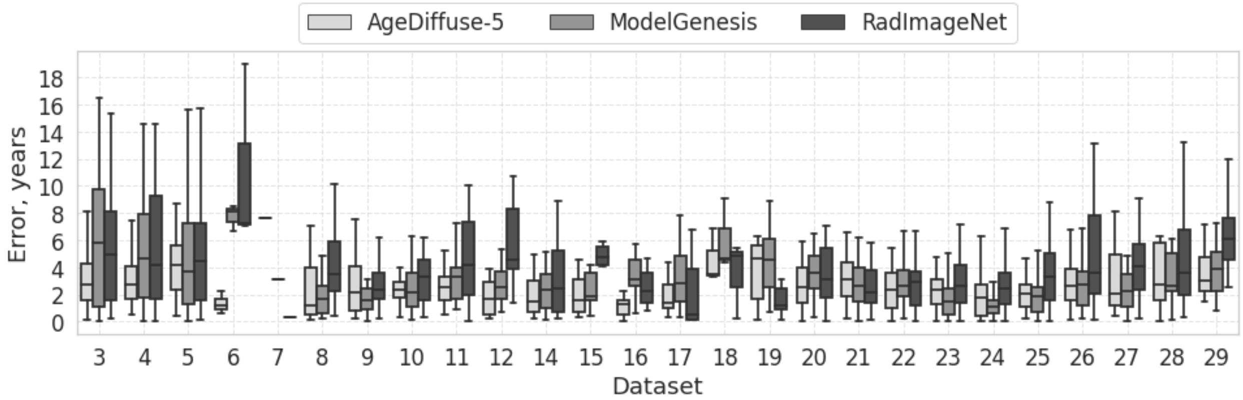

Figure S1 Box plots for prediction error distribution of different models for each chronological age on Test Set 1(n=583) AgeDiffuse-5 demonstrated strong performance across the age range, with mild performance degradation for subjects older than 25 years.

A2. Linear model diagnostics

We analyzed linear model fit using the statsmodels python package to identify potential problems that can occur from fitting linear regression model to non-linear relation.

Firstly, we compare three linear regression with VV, WMV, sGMV, GMV and sex variables as predictors, and 2D RadImageNet, 3D ModelGenesis and 2D AgeDiffuse-1 predicted brain age versus chronological age on the overlapping test set 1 (see Table S1). We used the centile definition described in Bethlehem et al (Bethlehem et al., 2022); We obtained a total of 372 overlapping scans for test set 1 from datasets IXI(*IXI Dataset – Brain Development*, n.d.), Pixar(*MRI Data of 3-12 Year Old Children and Adults during Viewing of a Short Animated Film*, n.d.), SALD(Wei et al., 2018).

Table S1. Linear model diagnostics analysis – comparison of VV, WMV, sGMV, GMV and sex variables as predictors, and 2D RadImageNet, 3D ModelGenesis and 2D AgeDiffuse-1 predicted brain age versus chronological age on the overlapping test set 1(N=372).

	R2	F-statistics	AIC
2D RadImageNet	0.725	192.6	2027
3D ModelGenesis	0.778	256.9	2018
**2D AgeDiffuse-1**	**0.772**	**277.5**	**1991**
**2D AgeDiffuse-5**	**0.791**	**247.5**	**1933**
Chronological Age	0.783	264.1	2074

Using pairwise one-sided F-statistics we found a significant difference in variance between the 2D RadImageNet and 2D AgeDiffuse-1 model predictions (p=6.5e-8, see Table S2). There are no significant differences in variance between the 3D ModelGenesis model and any of the other models, based on the Bonferroni corrected p-values all being > 0.0005.

Finally, we compared two linear regressions with VV, WMV, sGMV, GMV and sex variables as predictors and chronological age versus predicted brain age using the best model AgeDiffuse-5 as dependent variables for all overlapping subjects (N=25,090) and found that the brain age variable had a higher R-squared value (R2:0.37, F-stat:2936, AIC:1.1e+5, Figure S6 vs R2:0.48, F-stat:4587, AIC: 1.1e+5, Figure S7), indicating a stronger correlation between structural changes and predicted brain age as compared to chronological age.

Table S2 Pairwise F-statistics measures(one-way, with p-value in parenthesis). If the p-value is less than the significance level, then the null hypothesis can be rejected, and it can be concluded that the variances of the two data sets are different. P values were adjusted for multiple comparisons using the Bonferroni correction. Significant differences (with corrected P < 0.0005) are highlighted with an asterisk.

	2D RadImageNet	3D ModelGenesis	2D AgeDiffuse-1	2D AgeDiffuse-5	Chronological Age
2D RadImageNet	-	9.5(0.002)	**29.8(6.5e-8)***	9.1(0.003)	5.0(0.02)
3D ModelGenesis	-	-	4.2(0.04)	0.05(0.8)	0.44(0.5)
2D AgeDiffuse-1	-	-	-	5.9(0.02)	7.03(0.008)
2D AgeDiffuse-5	-	-	-	-	0.22(0.64)

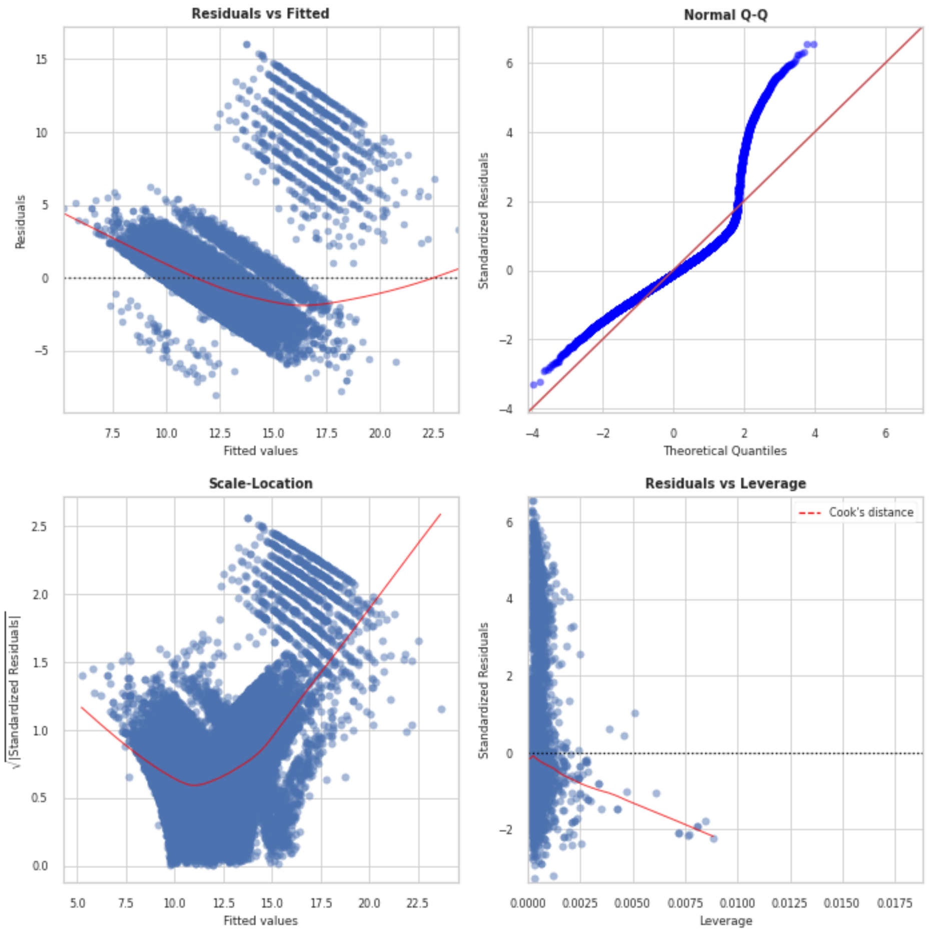

Figure S2 Chronological Age Linear Model Diagnostics(R2:0.37, F-stat:2936, AIC:1.1e+5). ***Top left:*** *Residual vs Fitted values. In the graph, a red (roughly) horizontal line would be an indicator that the residual has a linear pattern.* ***Top right:*** *Standardized Residual vs Theoretical Quantile to check if residuals are normally distributed visually.* ***Bottom left:*** *Sqrt(Standardized Residual) vs Fitted values to check homoscedasticity of the residuals, with non-horizontal scatter suggesting the variance of errors is not constant.* ***Bottom right:*** *Residual vs Leverage Points falling outside the Cook's distance curves are considered observations that can sway the fit.*

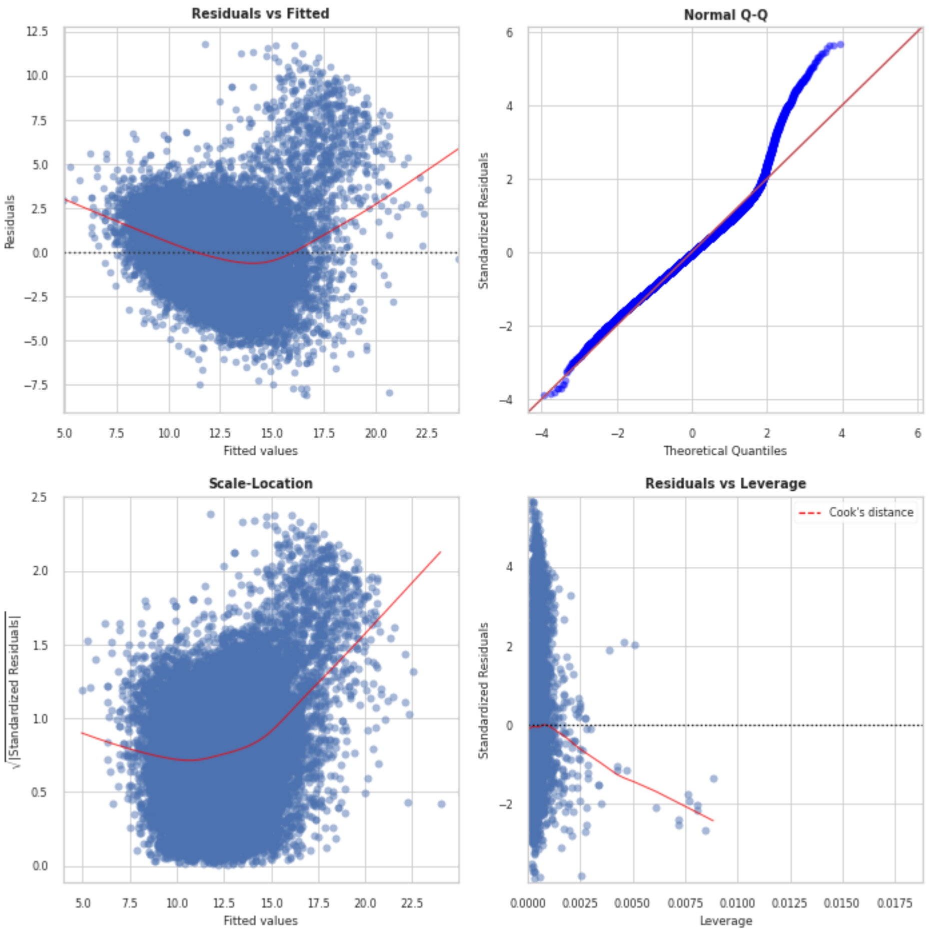

Figure S3 Predicted Brain Age Linear Model Diagnostics(R2:0.48, F-stat:4587, AIC: 1.1e+5). ***Top left:*** *Residual vs Fitted values. In the graph, a red (roughly) horizontal line would be an indicator that the residual has a linear pattern.* ***Top right:*** *Standardized Residual vs Theoretical Quantile to check if residuals are normally distributed visually.* ***Bottom left:*** *Sqrt(Standardized Residual) vs Fitted values to check homoscedasticity of the residuals, with non-horizontal scatter suggesting the variance of errors is not constant.* ***Bottom right:*** *Residual vs Leverage Points falling outside the Cook's distance curves are considered observations that can sway the fit.*

A3. Age-Bias Correction

We used the linear bias correction method described by Smith et al. (Smith et al., 2019) for bias correction for the gap. Such a bias correction is valuable for most brain-age prediction studies, as there is normally an underfitting of the prediction due to problems such as regression dilution and non-Gaussian age distribution. Defining y to be chronological age and x the predicted age, we fitted a linear regression $x=ay+b$ to the left-out validation set (with labels). The corrected predicted age is estimated by $x=(x-b)/a$ This method requires (at the point of estimating a and b from x and y) that the chronological ages are known. For the two external test sets, we assumed that a and b are generalizable. We used the coefficients (a=1.1, b=-2.2) fitted on Test Set 1 to estimate the corrected brain-age gap (Figure 4). We found that brain age correction does not improve MAE on Test Set 2 (no correction MAE = 1.9 years; with correction MAE = 2.6 years). We hypothesize that the brain age correction procedure does not generalize well on unseen datasets and does not capture the non-linear, complex relationship between brain age and chronological age, unlike deep learning, and therefore we used “raw” brain age predictions for all the analysis in this paper.

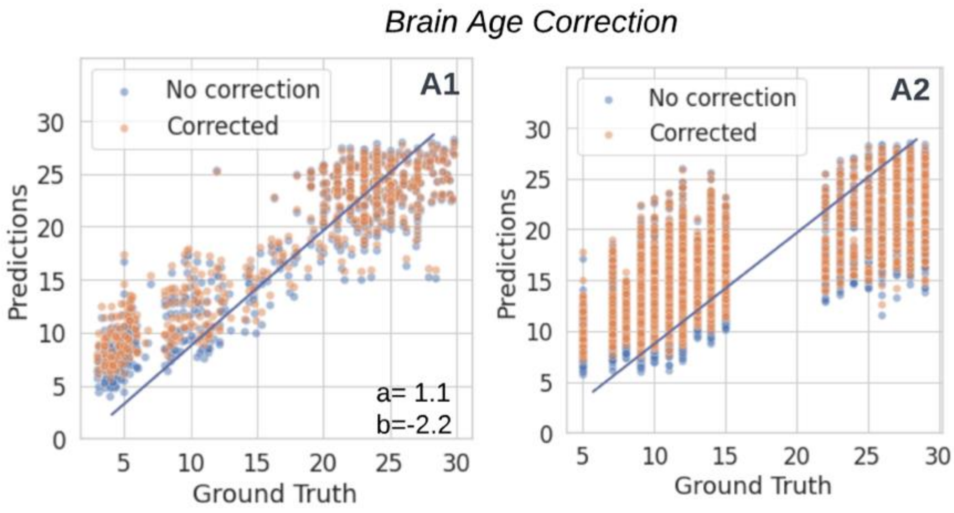

Figure S4 Brain age-correction scatterplots with fitted regression line. Panel A1: Test Set 1(N=583), Panel A2: Test Set 2 (N=27.719). We fitted linear regression using Test Set1, a=1.1, b=-2.2.

A4. Outlier Analysis

To investigate performance drop in the WU1200 dataset, we compared key volumetric measures in mm^3(VV, WMV, sGMV, GMV) between WU1200 (N=620) and an adult test set (age 22+, N=226; datasets: IXI(*IXI Dataset – Brain Development*, n.d.), Pixar(*MRI Data of 3-12 Year Old Children and Adults during Viewing of a Short Animated Film*, n.d.), SALD(Wei et al., 2018)). We used pairwise Mann-Whitney-U test and calculated the adjusted alpha to account for multiple comparisons using Bonferroni correction (adjusted P=0.0125). We also calculate Cohen's d effect size to quantify the standardized mean difference. We found that VV (Cohen's d=0.98), sGMV (Cohen's d=0.25) were significantly higher and WMV (Cohen's d=-0.49) was significantly lower in WU1200. This could indicate developmental differences, highlighting that the observed performance drop may be due more to true population differences than problems with the model.

A5. Primary Datasets

Table S3 Dataset demographics

Dataset	Split	Age years, median [min,max]	Sex, F %	Number MRIs
ABIDE	Train	11 [7,35]	35%	369
AOMIC	Train	22 [19,26]	52%	928
Calgary	Train	4 [3,6]	43%	249
ICBM	Train	25 [18,35]	46%	808
NIMH	Train	11 [4,22]	53%	917
PING	Train	11 [3,21]	49%	738
Healthy adults(HAN)	Train	26 [18,35]	65%	318
Petfrog	Train	21 [12,33]	52%	268
Pixar	Test1	6 [4,34]	61%	126
Baby Connectome	Test1	4 [3,7]	49%	70
IXI	Test1	25 [20,30]	56%	101
SALD	Test1	24 [19,29]	63%	151
NYU2(CoRR)	Test1	15 [7,28]	57%	135
ABCD	Test2 & Long Test	10[8,15]	47%	26814
Long579	Test2	7[5,9]	61%	285
WU1200	Test2	26[22,29]	46%	620
28andme	Long Test	23 [23-23]	100%	60

ABCD

Data used in the preparation of this article were obtained from the Adolescent Brain Cognitive Development SM (ABCD) Study (https://abcdstudy.org), held in the NIMH Data Archive (NDA). This is a multisite, longitudinal study designed to recruit more than

10,000 children age 9-10 and follow them over 10 years into early adulthood. The ABCD Study® is supported by the National Institutes of Health and additional federal partners under award numbers U01DA041048, U01DA050989, U01DA051016, U01DA041022, U01DA051018, U01DA051037, U01DA050987, U01DA041174, U01DA041106, U01DA041117, U01DA041028, U01DA041134, U01DA050988, U01DA051039, U01DA041156, U01DA041025, U01DA041120, U01DA051038, U01DA041148, U01DA041093, U01DA041089, U24DA041123, U24DA041147. A full list of supporters is available at https://abcdstudy.org/federal-partners.html. A listing of participating sites and a complete listing of the study investigators can be found at https://abcdstudy.org/consortium_members/. ABCD consortium investigators designed and implemented the study and/or provided data but did not necessarily participate in the analysis or writing of this report. This manuscript reflects the authors' views and may not reflect the opinions or views of the NIH or ABCD consortium investigators.

The ABCD data repository grows and changes over time. The ABCD data used in this report came from the fast-track data release. The raw data are available at https://nda.nih.gov/edit_collection.html?id=2573. Instructions on how to create an NDA study are available at https://nda.nih.gov/training/modules/study.html).

Additional support for this work was made possible from supplements to U24DA041123 and U24DA041147, the National Science Foundation (NSF 2028680), and Children and Screens: Institute of Digital Media and Child Development Inc. (Casey et al., 2018)

ABIDE

ABIDE II involves 19 sites, ten charter institutions and seven new members, overall donating 1114 datasets from 521 individuals with ASD and 593 controls (age range: 5-64 years). These data were openly released to the scientific community on June 2016. In accordance with HIPAA guidelines and 1000 Functional Connectomes Project / INDI protocols, all datasets are anonymous, with no protected health information included. Consistent with its popularity in the imaging community and prior usage in FCP/INDI efforts, the NIFTI format was selected to store the ABIDE II MRI datasets (http://fcon_1000.projects.nitrc.org/indi/abide/abide_II.html). With the exception of a single collection (IP1, 1.5 Tesla), all MRI data were acquired using 3 Tesla scanners(Di Martino et al., 2017).

Aomic

The Amsterdam Open MRI Collection (AOMIC, https://openneuro.org/datasets/ds003097/versions/1.2.1) is a collection of three datasets with multimodal (3T) MRI data, including structural (T1-weighted), diffusion-weighted, and (resting-state and task-based) functional BOLD MRI data, as well as detailed demographics and psychometric variables from a large set of healthy participants (N = 928, N = 226, and N = 216). Data from all three datasets were acquired on the same Philips 3T scanner (Philips, Best, the Netherlands) but underwent several upgrades in between the three studies (Snoek et al., 2021).

Baby Connectome

The Baby Connectome Project (BCP: https://nda.nih.gov/edit_collection.html?id=2848) is a four-year study of children from birth through five years of age, intended to provide a better understanding of how the brain develops from infancy through early childhood and the factors that contribute to healthy brain development. This project is a research initiative of the Neuroscience Blueprint – a cooperative effort among the 15 NIH Institutes, Centers, and Offices that support neuroscience research. The BCP is supported by Wyeth Nutrition through a donation to the FNIH. Images are acquired on 3T Siemens Prisma MRI scanners using a Siemens 32-channel head coil at the Center for Magnetic Resonance Research (CMRR) at the University of Minnesota and the Biomedical Research Imaging Center (BRIC) at the University of North Carolina at Chapel Hill (Howell et al., 2019).

Calgary

The Preschool MRI study in The Developmental Neuroimaging Lab at the University of Calgary uses different magnetic resonance imaging (MRI) techniques to study brain structure and function in early childhood (https://osf.io/axz5r/files/osfstorage). All imaging for this dataset was conducted using the same General Electric 3T MR750w system and 32-channel head coil (GE, Waukesha, WI) at the Alberta Children's Hospital in Calgary, Canada. Children were scanned either while awake and watching a movie, or while sleeping without sedation. The University of Cal- gary Conjoint Health Research Ethics Board (CHREB) approved this study (REB13-0020). T1-weighted images were acquired using an FSPGR BRAVO sequence with TR = 8.23 ms, TE = 3.76 ms, TI = 540 ms, flip angle=12 degrees, voxel size = 0.9x0.9x0.9 mm3, 210 slices,

matrix size=512x512, field of view=23.0 cm. ASL images were acquired with the vendor supplied pseudo continuous 3D ASL sequence with TR = 4.56 s, TE = 10.7 ms, in-plane reso- lution of 3.5x3.5 mm2, post label delay of 1.5 s, and thirty 4.0 mm thick slices. The sequence scan time was 4.4 minutes (Reynolds et al., 2020)

ICBM

Data used in the preparation of this work were obtained from the International Consortium for Brain Mapping (ICBM) database (www.loni.usc.edu/ICBM). The ICBM project (Principal Investigator John Mazziotta, M.D., University of California, Los Angeles) is supported by the National Institute of Biomedical Imaging and BioEngineering. ICBM is the result of efforts of co-investigators from UCLA, Montreal Neurologic Institute, University of Texas at San Antonio, and the Institute of Medicine, Juelich/Heinrich Heine University - Germany. Data collection and sharing for this project was provided by the International Consortium for Brain Mapping (ICBM; Principal Investigator: John Mazziotta, MD, PhD). ICBM funding was provided by the National Institute of Biomedical Imaging and BioEngineering. ICBM data are disseminated by the Laboratory of Neuro Imaging at the University of Southern California (Kötter et al., 2001).

IXI

The data has been collected at three different hospitals in London:Hammersmith Hospital using a Philips 3T system (details of scanner parameters: http://brain-development.org/scanner-philips-medical-systems-intera-3t/), Guy's Hospital using a

Philips 1.5T system (details of scanner parameters: http://brain-development.org/scanner-philips-medical-systems-gyroscan-intera-1-5t/), Institute of Psychiatry using a GE 1.5T system (details of the scan parameters not available at the moment). The Thames Valley MREC granted ethical approval. The T1 and T2 images were acquired prior to diffusion-weighted imaging using 3D MRPRAGE and dual-echo weighted imaging (*IXI Dataset – Brain Development*, n.d.).

NIMH

The data used in this work was collected from the 5.1 release (https://nda.nih.gov/edit_collection.html?id=1151) . MRI scans were acquired using either General Electric or Siemens 1.5 Tesla scanners involving six sites or Pediatric Study Centers (PSC) in the United States. The Institutional Review Board at the University of Wisconsin-Madison also approved the analysis of the data of this human subject. Sequence type: 3D FLASH/SPGR; GE sequence: pulse sequence=SPGR, mode=3D; TR: 22 ms; TE: 10-11 ms; excitation pulse angle: 30 degrees; orientation: sagittal; FoV: 250mmISx250mmAP; matrix: 256 x 256 (x 124 - 180 slices); slices: 160-180 slices of 1-1.5 mm thickness (cover entire head). Note that on GE systems with a 124-slice limitation, slice thickness should be adjusted to cover the entire head with 124 slices: signal averages: 1; scan time: 11.6 – 16.8 min (Evans, 2006).

PING

The PING Data Resource(https://nda.nih.gov/edit_collection.html?id=2607) is the product of a multi-site project involving developmental researchers across the United States, including UC San Diego, the University of Hawaii UC Los Angeles Children's Hospital of Los Angeles of the University of Southern California UC Davis Kennedy Krieger Institute of Johns Hopkins University Sackler Institute of Cornell University University of Massachusetts Massachusetts General Hospital at Harvard University and Yale University. The Data Resource includes neurodevelopmental histories, information about developing mental and emotional functions, multimodal brain imaging data, and genotypes for well over 1000 children and adolescents between the ages of 3 and 20. The PING imaging protocol takes advantage of key technologies developed for the consortium and builds on earlier methods development performed as part of the Biomedical Informatics Research Network (BIRN (Keator et al., 2008) and the Alzheimer's Disease Neuroimaging Initiative (ADNI (*The Alzheimer's Disease Neuroimaging Initiative (ADNI): MRI Methods - Jack - 2008 - Journal of Magnetic Resonance Imaging - Wiley Online Library*, n.d.)). Specifically, a standard PING scan session included: 1) a 3D T1-weighted inversion prepared RF-spoiled gradient echo scan using prospective motion correction (PROMO), for cortical and subcortical segmentation; 2) a 3D T2-weighted variable flip angle fast spin echo scan, also using PROMO, for detection and quantification of white matter lesions and segmentation of VV; 3) a high angular resolution diffusion imaging (HARDI) scan, with integrated B0 distortion correction (DISCO), for segmentation of white matter tracts and measurement of diffusion parameters; and 4) a resting state blood oxygenation level-dependent (BOLD) fMRI scan, with integrated distortion correction. Pulse sequence

parameters used across (3 T) scanner manufacturers (GE, Siemens, and Phillips) and models were optimized for equivalence in contrast properties and consistency in image-derived quantitative measures (Jernigan et al., 2016).

Pixar

One hundred twenty-two 3.5–12-year-old children (M(s.d.) = 6.7(2.3); 64 females) participated in the study (https://openfmri.org/dataset/ds000228/). Child and adult participants were recruited from the local community. All adult participants gave written consent; parent/guardian consent and child assent was received for all child participants. Recruitment and experiment protocols were approved by the Committee on the Use of Humans as Experimental Subjects (COUHES) at the Massachusetts Institute of Technology. Whole-brain structural and functional MRI data were acquired on a 3-Tesla Siemens Tim Trio scanner located at the Athinoula A. Martinos Imaging Center at MIT. Children under age 5 years used one of two custom 32-channel phased-array head coils made for younger (n = 3, M(s.d.) = 3.91(.42) years) or older (n = 28, M(s.d.) = 4.07(.42) years) children; all other participants used the standard Siemens 32-channel head coil. T1-weighted structural images were collected in 176 interleaved sagittal slices with 1 mm isotropic voxels (GRAPPA parallel imaging, acceleration factor of 3; adult coil: FOV: 256 mm; kid coils: FOV: 192 mm). Functional data were collected with a gradient-echo EPI sequence sensitive to Blood Oxygen Level Dependent (BOLD) contrast in 32 interleaved near-axial slices aligned with the anterior/posterior commissure and covering the whole brain (EPI factor: 64; TR: 2 s, TE: 30 ms, flip angle: 90°). This data was obtained from the OpenfMRI database, accession

number is ds000228. Dataset version 1.0.2 (*MRI Data of 3-12 Year Old Children and Adults during Viewing of a Short Animated Film*, n.d.)

SALD

The data was generated in the Southwest University Adult Lifespan Dataset (SALD), which comprises a large cross-sectional sample (n = 494; age range = 19-80) undergoing a multi-modal (sMRI, rs-fMRI, and behavioral). All data were collected at the Southwest University Center for Brain Imaging using a 3.0-T Siemens Trio MRI scanner (Siemens Medical, Erlangen, Ger- many). A magnetization-prepared rapid gradient echo (MPRAGE) sequence was used to acquire high-resolution T1-weighted anatomical images (repetition time=1,900ms, echo time=2.52ms, inversion time=900ms, flip angle=90 degrees, resolution matrix=256×256, slices=176, thickness =1.0mm, and voxel size=111mm3) (Wei et al., 2018).

NYU2(CoRR)

The Consortium for Reliability and Reproducibility (CoRR, http://fcon_1000.projects.nitrc.org/fcpClassic/FcpTable.html) has aggregated 1,629 typical individuals' resting state fMRI (rfMRI) data (5,093 rfMRI scans) from 18 international sites and is openly sharing them via the International Data-sharing Neuroimaging Initiative (INDI). In this study, we used a subset from CoRR study "NYU 2" created by New York University (Di Martino, Kelly)(*An Open Science Resource for Establishing Reliability and Reproducibility in Functional Connectomics* | *Scientific Data*, n.d.).

Healthy adults

The dataset was collected and shared under the NIMH Healthy Research Volunteer (RV) Study (Recruitment and Characterization of Healthy Research Volunteer for NIMH Intramural Studies NCT033046, https://openneuro.org/datasets/ds004215/versions/1.0.1). Data collection is ongoing, while data from 1,090 participants (155 with MRI) is shared. The MR protocol used was initially based on the ADNI-3 basic protocol, but was later modified to include portions of the ABCD protocol. Because there may be small changes in parameters from the standard ABCD/ADNI3 sequences, detailed sequence descriptions are shared in the BIDS source data directory. (Nugent et al., 2022).

28andMe

In this set of dense-sampling, deep phenotyping studies, we determined whether day-to-day variation in sex hormone concentrations impacts large-scale brain network connectivity. In Study 1 (sessions 1-30, 2018), the female participant was naturally cycling; in Study 2 (sessions 31-60, 2019), the participant was placed on an oral hormonal contraceptive regimen. The participant underwent a daily magnetic resonance imaging scan on a Siemens 3T Prisma scanner equipped with a 64-channel phased-array head coil. First, high-resolution anatomical scans were acquired using a T1-weighted magnetization prepared rapid gradient echo (MPRAGE) sequence (TR = 2500 ms, TE = 2.31 ms, TI = 934 ms, flip angle = 7º, 0.8 mm thickness) followed by a gradient echo fieldmap (TR = 758 ms; TE1 = 4.92 ms; TE2 = 7.38 ms; flip angle = 60º).

Next, the participant completed a 10-minute resting-state fMRI scan using a T2*-weighted multi-band echo-planar imaging (EPI) sequence sensitive to the blood oxygenation level-dependent (BOLD) contrast (72 oblique slices, TR = 720 ms, TE = 37 ms, voxel size = 2 mm3, flip angle = 56º, multiband factor = 8). High-resolution anatomical scans were acquired using a T1-weighted magnetization prepared rapid gradient echo (MPRAGE) sequence (TR = 2500 ms, TE = 2.31 ms, TI = 934 ms, flip angle = 7º, 0.8 mm thickness) followed by a gradient echo fieldmap (TR = 758 ms; TE1 = 4.92 ms; TE2 = 7.38 ms; flip angle = 60º). A T2- weighted turbo spin echo (TSE) scan was also acquired with an oblique coronal orientation positioned orthogonally to the main axis of the hippocampus (TR/TE= 8100/50 ms, flip angle = 122°, 0.4 × 0.4 mm2 in plane resolution, 2 mm slice thickness, 31 interleaved slices with no gap, total acquisition time = 4:21 min) (Pritschet et al., 2020).

Long579

The public neuroimaging and behavioral dataset entitled “A longitudinal neuroimaging dataset on language processing in children ages 5, 7, and 9 years old” available on the OpenNeuro project (https://openneuro.org) and organized in compliance with the Brain Imaging Data Structure (BIDS). It includes 322 participants, recruited from the Austin, Texas. All neuroimaging data were collected using a Siemens Skyra 3 T MRI scanner located at The University of Texas at Austin Imaging Research Center. All images were acquired using a 64-channel head coil. Participants were positioned supine in the MRI scanner and foam pads were placed around the head to minimize movement. T1-weighted Magnetization Prepared - RApid Gradient Echo (MPRAGE) images were

collected using GRAPPA, a parallel imaging technique based on k-space, and the following parameters: GRAPPA accel.factor PE = 2, TR = 1900 ms, TE = 2.43 ms, field of view = 256 mm, matrix size = 256 × 256, bandwidth = 180 Hz/Px, slice thickness = 1 mm, number of slices = 192, voxel size = 1 mm isotropic, flip angle = 9°. (Wang et al., 2022)

WU1200

This HCP data release includes high-resolution 3T MR scans from young healthy adult twins and non-twin siblings (ages 22-35) using four imaging modalities: structural images (T1w and T2w), resting-state fMRI (rfMRI), task-fMRI (tfMRI), and high angular resolution diffusion imaging (dMRI). Behavioral and other individual subject measure data (both NIH Toolbox and non-Toolbox measures) is available on all subjects. MEG data and 7T MR data is available for a subset of subjects (twin pairs). The Open Access Dataset includes imaging data and most behavioral data. All details in the imaging protocols can be found at study webpage (https://humanconnectome.org/study/hcp-young-adult/document/1200-subjects-data-release/)

References

An open science resource for establishing reliability and reproducibility in functional connectomics | Scientific Data. (n.d.). Retrieved February 15, 2023, from https://www.nature.com/articles/sdata201449

Bethlehem, R. a. I., Seidlitz, J., White, S. R., Vogel, J. W., Anderson, K. M., Adamson, C., Adler, S., Alexopoulos, G. S., Anagnostou, E., Areces-Gonzalez, A., Astle, D. E., Auyeung, B., Ayub, M., Bae, J., Ball, G., Baron-Cohen, S., Beare, R., Bedford, S. A., Benegal, V., … Alexander-Bloch, A. F. (2022). Brain charts for the human lifespan. *Nature*, *604*(7906), Article 7906. https://doi.org/10.1038/s41586-022-04554-y

Casey, B. J., Cannonier, T., Conley, M. I., Cohen, A. O., Barch, D. M., Heitzeg, M. M., Soules, M. E., Teslovich, T., Dellarco, D. V., Garavan, H., Orr, C. A., Wager, T. D., Banich, M. T., Speer, N. K., Sutherland, M. T., Riedel, M. C., Dick, A. S., Bjork, J. M., Thomas, K. M., … ABCD Imaging Acquisition Workgroup. (2018). The Adolescent Brain Cognitive Development (ABCD) study: Imaging acquisition across 21 sites. *Developmental Cognitive Neuroscience*, *32*, 43–54. https://doi.org/10.1016/j.dcn.2018.03.001

Di Martino, A., O'Connor, D., Chen, B., Alaerts, K., Anderson, J. S., Assaf, M., Balsters, J. H., Baxter, L., Beggiato, A., Bernaerts, S., Blanken, L. M. E., Bookheimer, S. Y., Braden, B. B., Byrge, L., Castellanos, F. X., Dapretto, M., Delorme, R., Fair, D. A., Fishman, I., … Milham, M. P. (2017). Enhancing studies of the connectome in autism using the autism brain imaging data exchange II. *Scientific Data*, *4*(1), Article 1. https://doi.org/10.1038/sdata.2017.10

Evans, A. C. (2006). The NIH MRI study of normal brain development. *NeuroImage*, *30*(1), 184–202. https://doi.org/10.1016/j.neuroimage.2005.09.068

Howell, B. R., Styner, M. A., Gao, W., Yap, P.-T., Wang, L., Baluyot, K., Yacoub, E., Chen, G., Potts, T., Salzwedel, A., Li, G., Gilmore, J. H., Piven, J., Smith, J. K., Shen, D., Ugurbil, K., Zhu, H., Lin, W., & Elison, J. T. (2019). The UNC/UMN Baby Connectome Project (BCP): An overview of the study design and protocol development. *NeuroImage*, *185*, 891–905. https://doi.org/10.1016/j.neuroimage.2018.03.049

IXI Dataset – Brain Development. (n.d.). Retrieved February 15, 2023, from https://brain-development.org/ixi-dataset/

Jernigan, T. L., Brown, T. T., Hagler, D. J., Akshoomoff, N., Bartsch, H., Newman, E., Thompson, W. K., Bloss, C. S., Murray, S. S., Schork, N., Kennedy, D. N., Kuperman, J. M., McCabe, C., Chung, Y., Libiger, O., Maddox, M., Casey, B. J., Chang, L., Ernst, T. M., … Dale, A. M. (2016). The Pediatric Imaging, Neurocognition, and Genetics (PING) Data Repository. *NeuroImage*, *124*, 1149–1154. https://doi.org/10.1016/j.neuroimage.2015.04.057

Keator, D. B., Grethe, J. S., Marcus, D., Ozyurt, B., Gadde, S., Murphy, S., Pieper, S., Greve, D., Notestine, R., Bockholt, H. J., & Papadopoulos, P. (2008). A National Human Neuroimaging Collaboratory Enabled by the Biomedical Informatics Research Network (BIRN). *IEEE Transactions on Information Technology in Biomedicine*, *12*(2), 162–172. https://doi.org/10.1109/TITB.2008.917893

Kötter, R., Mazziotta, J., Toga, A., Evans, A., Fox, P., Lancaster, J., Zilles, K., Woods, R., Paus, T., Simpson, G., Pike, B., Holmes, C., Collins, L., Thompson, P., MacDonald, D., Iacoboni, M., Schormann, T., Amunts, K., Palomero-Gallagher, N., … Mazoyer, B. (2001). A probabilistic atlas and reference system for the human brain: International Consortium for Brain Mapping (ICBM). *Philosophical Transactions of the Royal Society*

of London. Series B: Biological Sciences, *356*(1412), 1293–1322. https://doi.org/10.1098/rstb.2001.0915

MRI data of 3-12 year old children and adults during viewing of a short animated film. (n.d.). Retrieved February 15, 2023, from https://openfmri.org/dataset/ds000228/

Nugent, A. C., Thomas, A. G., Mahoney, M., Gibbons, A., Smith, J. T., Charles, A. J., Shaw, J. S., Stout, J. D., Namyst, A. M., Basavaraj, A., Earl, E., Riddle, T., Snow, J., Japee, S., Pavletic, A. J., Sinclair, S., Roopchansingh, V., Bandettini, P. A., & Chung, J. (2022). The NIMH intramural healthy volunteer dataset: A comprehensive MEG, MRI, and behavioral resource. *Scientific Data*, *9*(1), Article 1. https://doi.org/10.1038/s41597-022-01623-9

Pritschet, L., Santander, T., Taylor, C. M., Layher, E., Yu, S., Miller, M. B., Grafton, S. T., & Jacobs, E. G. (2020). Functional reorganization of brain networks across the human menstrual cycle. *NeuroImage*, *220*, 117091. https://doi.org/10.1016/j.neuroimage.2020.117091

RadImageNet: An Open Radiologic Deep Learning Research Dataset for Effective Transfer Learning | Radiology: Artificial Intelligence. (n.d.). Retrieved August 17, 2023, from https://pubs.rsna.org/doi/full/10.1148/ryai.210315

Reynolds, J. E., Long, X., Paniukov, D., Bagshawe, M., & Lebel, C. (2020). Calgary Preschool magnetic resonance imaging (MRI) dataset. *Data in Brief*, *29*, 105224. https://doi.org/10.1016/j.dib.2020.105224

Smith, S. M., Vidaurre, D., Alfaro-Almagro, F., Nichols, T. E., & Miller, K. L. (2019). Estimation of brain age delta from brain imaging. *NeuroImage*, *200*, 528–539. https://doi.org/10.1016/j.neuroimage.2019.06.017

Snoek, L., van der Miesen, M. M., Beemsterboer, T., van der Leij, A., Eigenhuis, A., & Steven Scholte, H. (2021). The Amsterdam Open MRI Collection, a set of multimodal MRI datasets for individual difference analyses. *Scientific Data*, *8*(1), Article 1. https://doi.org/10.1038/s41597-021-00870-6

The Alzheimer's disease neuroimaging initiative (ADNI): MRI methods—Jack—2008—Journal of Magnetic Resonance Imaging—Wiley Online Library. (n.d.). Retrieved April 12, 2023, from https://onlinelibrary.wiley.com/doi/full/10.1002/jmri.21049

Wang, J., Lytle, M. N., Weiss, Y., Yamasaki, B. L., & Booth, J. R. (2022). A longitudinal neuroimaging dataset on language processing in children ages 5, 7, and 9 years old. *Scientific Data*, *9*(1), Article 1. https://doi.org/10.1038/s41597-021-01106-3

Wei, D., Zhuang, K., Ai, L., Chen, Q., Yang, W., Liu, W., Wang, K., Sun, J., & Qiu, J. (2018). Structural and functional brain scans from the cross-sectional Southwest University adult lifespan dataset. *Scientific Data*, *5*(1), Article 1. https://doi.org/10.1038/sdata.2018.134

Zhou, Z., Sodha, V., Siddiquee, M. M. R., Feng, R., Tajbakhsh, N., Gotway, M. B., & Liang, J. (2019). Models Genesis: Generic Autodidactic Models for 3D Medical Image Analysis. *Medical Image Computing and Computer-Assisted Intervention : MICCAI ... International Conference on Medical Image Computing and Computer-Assisted Intervention*, *11767*, 384–393. https://doi.org/10.1007/978-3-030-32251-9_42
